# Results for Two-Level Designs with General Minimum Lower-Order Confounding

**DOI:** 10.1155/2015/163234

**Published:** 2015-06-16

**Authors:** Zhi Ming Li, Run Chu Zhang

**Affiliations:** ^1^School of Mathematical Sciences, Xinjiang University, Urumqi 830046, China; ^2^KLAS and School of Mathematics, Northeast Normal University, Changchun 130024, China; ^3^LPMC and School of Mathematical Sciences, Nankai University, Tianjin 300071, China

## Abstract

The general minimum lower-order confounding (GMC) criterion for two-level design not only reveals the confounding information of factor effects but also provides a good way to select the optimal design, which was proposed by Zhang et al. (2008). The criterion is based on the aliased effect-number pattern (AENP). Therefore, it is very important to study properties of AENP for two-level GMC design. According to the ordering of elements in the AENP, the confounding information between lower-order factor effects is more important than that of higher-order effects. For two-level GMC design, this paper mainly shows the interior principles to calculate the leading elements _1_^#^ *C*_2_ and _2_^#^ *C*_2_ in the AENP. Further, their mathematical formulations are obtained for every GMC 2^*n*−*m*^ design with *N* = 2^*n*−*m*^ according to two cases: (i) 5*N*/16 + 1 ≤ *n* < *N*/2 and (ii) *N*/2 ≤ *n* ≤ *N* − 1.

## 1. Introduction

To find optimal designs in a more elaborate and explicit manner under effect hierarchy principle, Zhang et al. [1] first introduced the aliased effect-number pattern (AENP) and proposed a new criterion of general minimum lower-order confounding (GMC) for two-level regular design. Further, they proved that all the classification patterns conducting the existing criteria, such as maximum resolution (MR) criterion [2], minimum aberration (MA) criterion [3], clear effects (CE) criterion [4], and maximum estimation capacity (MEC) criterion [5], can be expressed as different functions of the AENP so that it can be a basis to unify these criteria.

Through the AENP, we can get a deeper understanding of properties of the above criteria and relationships among them. Zhang and Cheng [6] revealed an exact expression of the average minimum lower-order confounding property of MA design. Hu and Zhang [7] obtained an essential statistical equivalence of MEC design and MA design. From the average least confounding property between lower-order effects, MA designs are most suitable for the situation that all the factors in experiments are treated to be equally important, while GMC design has an individual least confounding property between lower-order effects and possesses the maximum numbers of clear main effects and clear two-factor interactions (2fi's). Because of this, GMC designs can be applied to the experiments which the experimenters have some prior information to the order of the importance factors. In practice, the latter situation more often happens than the former one. Therefore, the study for GMC designs should be significantly important in both theory and application.

Now we review some definitions proposed by Zhang et al. [1]. Let *D* be a 2^*n*−*m*^ design with *n* factors, *m* independent defining words, and *N* = 2^*n*−*m*^ runs. We denote the factors by 1,2,…, *n*. An *i*th-order factor effect is said to be aliased with *j*th-order factor effects at degree *k* if it is simultaneously aliased with *k* *j*th-order factor effects. The 0th-order effect is the grand mean and 1st-order effect is a main effect.

Let _*i*_^#^ *C*_*j*_^(*k*)^(*D*) (written by _*i*_^#^ *C*_*j*_^(*k*)^ for short) be the number of *i*th-order factor effects that are aliased with *k* *j*th-order factor effects. Denote $K_{j}=\left(\begin{array}{c} n\\ j \end{array}\right)$; a set {_*i*_^#^ *C*_*j*_^(*k*)^,  0 ≤ *k* ≤ *K*_*j*_,  0 ≤ *i*,  *j* ≤ *n*} is called* the aliased effect-number pattern (AENP)* of the design *D*. The set reflects the overall confounding between factor effects in the design. Define _*i*_^#^ *C*_*j*_ = (_*i*_^#^ *C*_*j*_^(0)^, _*i*_^#^ *C*_*j*_^(1)^,…, _*i*_^#^ *C*_*j*_^(*K*_*j*_)^) and a design that sequentially maximizes the vector

(1) C#=C1#2,C2#2,C1#3,C2#3,C3#2,C3#3,…

is called* a GMC design*, where the ordering of _*i*_^#^ *C*_*j*_'s is in accordance with the rule: _*i*_^#^ *C*_*j*_ *is before *_*u*_^#^ *C*_*v*_ *if either *max⁡(*i*, *j*) < max⁡(*u*, *v*),* or *max⁡(*i*, *j*) = max⁡(*u*, *v*),* with i* < *u*,* or *max⁡(*i*, *j*) = max⁡(*u*, *v*)* with i* = *u and j* < *v*. In order to make main effects or 2fi's estimable, we need to give an assumption:* the interactions involving three or more factors are absent*. Thus, we only study the leading terms _1_^#^ *C*_2_ and _2_^#^ *C*_2_ of AENP for two-level GMC design in this paper.

Zhang et al. [1] listed all two-level GMC designs of 16 and 32 runs, a number of 64-run GMC designs, and obtained the values of _1_^#^ *C*_2_ and _2_^#^ *C*_2_ by computer algorithm. However, the method is not suitable for designs with larger runs. Zhang and Cheng [6] and Chen and Liu [8] provided an important theory for constructing GMC designs. Cheng and Zhang [9] and Li et al. [10] finished the construction of GMC 2^*n*−*m*^ designs with *N*/4 + 1 ≤ *n* ≤ *N* − 1. However, there are few articles that pay attention to calculating the values of elements in the AENP, especially, the confounding information between main effects and 2fi's, or among 2fi's of two-level GMC design.

This paper mainly reveals the interior principles for calculating the values of _1_^#^ *C*_2_ and _2_^#^ *C*_2_ for two-level GMC design. In Section 2, we introduce some notations and obtain useful lemmas to study the lower-order confounding information of two-level GMC designs.  Section 3 and Section 4, respectively, obtain values of _1_^#^ *C*_2_ and _2_^#^ *C*_2_ for GMC 2^*n*−*m*^ design with resolution *R* ≥ *III*, for 5*N*/16 + 1 ≤ *n* < *N*/2 and *N*/2 ≤ *n* ≤ *N* − 1. Concluding remarks are given in Section 5.

## 2. Some Notations and Lemmas

Denote *q* = *n* − *m* and 1,2,…, *q* stand for *q* independent factors. Let *H*_*q*_ be the set containing all main effects 1,2,…, *q* and all interactions among them, formed by

(2) $$\begin{array}{c} H_{1}=\left\{1\right\},\;\;H_{q}=\left\{H_{q-1},q,qH_{q-1}\right\}, \end{array}$$

where *qH*_*q*−1_ = {*qd*: *d* ∈ *H*_*q*−1_}. By Theorem  2.7.1 of Mukerjee and Wu [11], any 2^*n*−*m*^ design *D* can be represented by an *n*-subset of *H*_*q*_; that is, *D* ⊂ *H*_*q*_.

Let *T*_1_ = *H*_1_ and *T*_*r*_ = {*r*, *rH*_*r*−1_} for 1 < *r* ≤ *q*. Evidently, *H*_*q*_ = ∪_*r*=1_^*q*^ *T*_*r*_. For 5*N*/16 + 1 ≤ *n* ≤ *N* − 1, Li et al. [10] have gotten that every GMC 2^*n*−*m*^ design is constructed by the last *n* columns of *H*_*q*_. Therefore, GMC 2^*n*−*m*^ designs with 5*N*/16 + 1 ≤ *n* < *N*/2 are directly formed by the last *n* columns of *T*_*q*_. Denote *S*_*qr*_ = *H*_*q*_∖*H*_*r*_ with 1 ≤ *r* < *q*. For *N*/2 ≤ *n* ≤ *N* − 1, there exists a number *r*  (<*q*) so that GMC 2^*n*−*m*^ design is formed by the last *n* columns of *T*_*r*_ ∪ *S*_*qr*_. Thus, the GMC design can be written by *D*_0_ ∪ *S*_*qr*_, where *D*_0_ consists of the last *n* − (*N* − 2^*r*^) columns of *T*_*r*_. To get the lower-order confounding information of two-level GMC design, we need to study structure of last *n*_0_ columns of *T*_*r*_ for *r* ≤ *q* and *n*_0_ ≤ *n*.

Suppose *D*_0_ consists of the last *n*_0_ columns of *T*_*r*_  (*r* ≤ *q*), where *n*_0_ = #{*D*_0_} and #*A* denotes the cardinality of a set *A*. The following example illustrates the structure of *D*_0_.

**Example 1 .**

Consider *r* = 7; we select the last *n*_0_ columns of *T*_7_ to construct *D*_0_. Clearly, there are 64 choices besides *D*_0_ ≡ *T*_*r*_. For 1 ≤ *n*_0_ ≤ 63, *D*_0_ is one of the following six forms.

- (*u* + 1) ⋯ 7*H*_*u*_ for 1 ≤ *u* < 7.
- (*u* + 1) ⋯ 7(*H*_*u*_∖*H*_*v*_) for 1 ≤ *v* < *u* < 7.
- (*u* + 1) ⋯ 7((*s* + 1) ⋯ *vH*_*s*_ ∪ (*H*_*u*_∖*H*_*v*_)) for 1 ≤ *s* < *v* < *u* < 7.
- (*u* + 1) ⋯ 7((*s* + 1) ⋯ *v*(*H*_*s*_∖*H*_*t*_)∪(*H*_*u*_∖*H*_*v*_)) for 1 ≤ *t* < *s* < *v* < *u* < 7.
- (*u* + 1) ⋯ 7((*s* + 1) ⋯ *v*((*w* + 1) ⋯ *tH*_*w*_ ∪ (*H*_*s*_∖*H*_*t*_))∪(*H*_*u*_∖*H*_*v*_)) for 1 ≤ *w* < *t* < *s* < *v* < *u* < 7.
- (*u* + 1) ⋯ 7((*s* + 1) ⋯ *v*((*w* + 1) ⋯ *t*(*H*_*w*_∖*H*_*z*_)∪(*H*_*s*_∖*H*_*t*_))∪(*H*_*u*_∖*H*_*v*_)) for 1 ≤ *z* < *w* < *t* < *s* < *v* < *u* < 7.

The above example provides a way to construct *D*_0_. Generally, for any *r*  (*r* ≤ *q*), we consider the construction of *D*_0_ in *T*_*r*_. Define

(3) Dt=Hit∖Hjt,  ait=it+1it+2⋯jt−1,               1≤t≤m,

where 1 ≤ *j*_*m*_ < *i*_*m*_ < *j*_*m*−1_ < *i*_*m*−1_ < ⋯<*j*_*t*_ < *i*_*t*_ < ⋯<*j*_1_ < *i*_1_ < *j*_0_ = *r*. Then, *D*_0_ can be constructed by either of the following cases.

**Case 1 .**

One has *D*_0_ = *a*_*i*_1__(*a*_*i*_2__(⋯(*a*_*i*_*m*−1__(*a*_*i*_*m*__ *D*_*m*_ ∪ *D*_*m*−1_)⋯) ∪ *D*_2_) ∪ *D*_1_).

**Case 2 .**

One has *D*_0_ = *a*_*i*_1__(*a*_*i*_2__(⋯(*a*_*i*_*m*−1__(*a*_*i*_*m*__ *H*_*i*_*m*__ ∪ *D*_*m*−1_)⋯) ∪ *D*_2_) ∪ *D*_1_).

In Case 1, the number of elements in *D*_0_ is even since #{*D*_0_} = ∑_*t*=1_^*m*^(2^*i*_*k*_^ − 2^*j*_*k*_^). However, that of *D*_0_ in Case 2 is odd because of #{*D*_0_} = ∑_*t*=1_^*m*−1^(2^*i*_*k*_^ − 2^*j*_*k*_^) + 2^*i*_*m*_^ − 1.

Consider *D* ⊂ *H*_*q*_ and any *γ* ∈ *H*_*q*_; define

(4) $$\begin{array}{c} B_{2}\left(D,\gamma \right)=\#\left\{\left(d_{1},d_{2}\right)\text{:}\;d_{1},d_{2}\in D,\;d_{1}d_{2}=\gamma \right\}, \end{array}$$

which is the number of 2fi's in *D* aliased with *γ*. By the definition of _*i*_^#^ *C*_*j*_^(*k*)^(*D*), it can be easily obtained that

(5) C1#2kD=#γ: γ∈D,B2D,γ=k,

(6) C2#2kD=k+1#γ: γ∈Hq,B2D,γ=k+1,

where *k* = 0,1,…, *K*_2_. In order to get the lower-order confounding of *D*_0_ in the above cases, we need to study *B*_2_(*D*_*t*_, *γ*) for *t* ≥ 1.

**Lemma 2 .**

Let *D*_*t*_ be defined in (3) for *t* ≥ 1. Then

(7) $$\begin{array}{c} B_{2}\left(D_{t},\gamma \right)=\left\{\begin{array}{cc} 2^{i_{t}-1}-2^{j_{t}-1}, & \gamma \in H_{j_{t}},\\ 2^{i_{t}-1}-2^{j_{t}}, & \gamma \in H_{i_{t}}\setminus H_{j_{t}},\\ 0, & \gamma \in H_{q}\setminus H_{i_{t}}. \end{array}\right. \end{array}$$

**Proof**

For *γ* ∈ *H*_*q*_∖*H*_*i*_*t*__, we have *B*_2_(*D*_*t*_, *γ*) = 0. If *γ* ∈ *H*_*j*_*t*__, then

(8) $$\begin{array}{c} B_{2}\left(D,\gamma \right)=\frac{\#\left\{H_{i_{t}}\setminus H_{j_{t}}\right\}}{2}=2^{i_{t}-1}-2^{j_{t}-1}. \end{array}$$

For *γ* ∈ *H*_*i*_*t*__∖*H*_*j*_*t*__, there are 2^*i*_*t*_−1^ − 1 pairs of factors in *H*_*i*_*t*__ so that their interactions are aliased with *γ*. Among these pairs, there are 2^*j*_*t*_^ − 1 pairs with one factor from *H*_*j*_*t*__ and another from *H*_*i*_*t*__∖*H*_*j*_*t*__. Thus,

(9) $$\begin{array}{c} B_{2}\left(D_{t},\gamma \right)=\left(2^{i_{t}-1}-1\right)-\left(2^{j_{t}}-1\right)=2^{i_{t}-1}-2^{j_{t}}. \end{array}$$

This completes the proof.

Next we analyze Case 1 of *D*_0_. For convenience, by (3), denote

(10) $$\begin{array}{c} D\left(t\right)=a_{i_{t}}\left(\cdots \left(a_{i_{m-1}}\left(a_{i_{m}}D_{m}\cup D_{m-1}\right)\cdots \right)\cup D_{t}\right) \end{array}$$

for 1 < *t* ≤ *m*. Evidently, *𝒟*(*t*) ⊂ *H*_*j*_*t*−1__ and *𝒟*(1) = *D*_0_ in Case 1. When *d*_1_ ∈ *𝒟*(*t*) and *d*_2_ ∈ *D*_*t*−1_, we have *d*_1_ *d*_2_ ∈ *D*_*t*−1_. Thus,

(11) $$\begin{array}{c} \#\left\{\left(d_{1},d_{2}\right)\text{:}\;d_{1}\in D\left(t\right),\;d_{2}\in D_{t-1},\;d_{1}d_{2}=\gamma \right\}=\overset{m}{\underset{l=t}{\sum }}\#\left\{D_{l}\right\} \end{array}$$

for *γ* ∈ *D*_*t*−1_. Otherwise, the value is zero. Then

(12) #d1,d2: d1∈Dt, d2∈Dt−1, d1d2=γ  =∑l=tm2il−2jl,γ∈Dt−1,0,γ∉Dt−1.

Based on Lemma 2 and (12), we can get the following result for Case 1.

**Lemma 3 .**

Let *D*_0_ = *a*_*i*_1__(*a*_*i*_2__(⋯(*a*_*i*_*m*−1__(*a*_*i*_*m*__ *D*_*m*_ ∪ *D*_*m*−1_)⋯) ∪ *D*_2_) ∪ *D*_1_). Then

(13) B2D0,γ=cm+12,γ∈Hjm,cm+1−ct+2it2,γ∈Dt,  t=1,…,m,ct2,γ∈Hjt−1∖Hit,  t=2,…,m,0,γ∈Hq∖Hi1,

where

(14) $$\begin{array}{c} c\left(1\right)=0,\;c\left(t\right)=\overset{t-1}{\underset{l=1}{\sum }}\left(2^{i_{l}}-2^{j_{l}}\right),\;t>1. \end{array}$$

**Proof**

For 1 < *l* ≤ *m*, by (10), we have

(15) B2ailDl∪Dl−1,γ =B2Dl∪Dl−1,γ =B2Dl,γ+B2Dl−1,γ  +#d1,d2: d1∈Dl, d2∈Dl−1, d1d2=γ.

Hence,

(16) B2D0,γ =∑l=1m−2B2Dl,γ+B2aimDm∪Dm−1,γ  +∑l=2m−1#d1,d2: d1∈Dl, d2∈Dl−1, d1d2=γ =∑l=1mB2Dl,γ  +∑l=2m#d1,d2: d1∈Dl, d2∈Dl−1, d1d2=γ.

Put *H*_*r*_ into 2*m* + 1 incompatible parts: *H*_*j*_*m*__, *D*_*l*+1_, and *H*_*j*_*l*__∖*H*_*i*_*l*+1__ for *l* = 0,1,…, *m* − 1. Clearly, if *γ* ∈ *H*_*q*_∖*H*_*i*_1__, then *B*_2_(*D*_0_, *γ*) = 0. By Lemma 2 and (12), we, respectively, discuss the following cases.

(i) If *γ* ∈ *H*_*j*_*m*__, then
  (17) $$\begin{array}{c} \#\left\{\left(d_{1},d_{2}\right)\text{:}\;d_{1}\in D\left(l\right),\;d_{2}\in D_{l-1},\;d_{1}d_{2}=\gamma \right\}=0 \end{array}$$
 for 1 < *l* ≤ *m* − 1. Thus,
  (18) $$\begin{array}{c} B_{2}\left(D_{0},\gamma \right)=\overset{m}{\underset{l=1}{\sum }}B_{2}\left(D_{t},\gamma \right)=\overset{m}{\underset{l=1}{\sum }}\left(2^{i_{l}-1}-2^{j_{l}-1}\right). \end{array}$$
(ii) If *γ* ∈ *D*_*t*_ with 1 < *t* ≤ *m*, one has
  (19) B2D0,γ =∑l=1t−1B2Dl,γ+B2Dt,γ  +#d1,d2: d1∈Dt+1, d2∈Dt, d1d2=γ =∑l=1t−12il−1−2jl−1+2it−1−2jt  +∑l=t+1m2il−2jl =∑l=1m2il−2jl−∑l=1t−12il−1−2jl−1−2it−1.
(iii) If *γ* ∈ *H*_*j*_*t*−1__∖*H*_*i*_*t*__ for *t* > 1, then
  (20) $$\begin{array}{c} B_{2}\left(D_{0},\gamma \right)=\overset{t-1}{\underset{l=1}{\sum }}B_{2}\left(D_{l},\gamma \right)=\overset{t-1}{\underset{l=1}{\sum }}\left(2^{i_{l}-1}-2^{j_{l}-1}\right). \end{array}$$

This completes the proof.

Lemma 3 shows that the value of *B*_2_(*D*_0_, *γ*) in Case 1 depends on all pairs {*i*_*t*_, *j*_*t*_}_1≤*t*≤*m*_ which relate to #{*D*_0_} = ∑_*t*=1_^*m*^(2^*i*_*t*_^ − 2^*j*_*t*_^). For instance, take *n*_0_ = #{*D*_0_} = 42 that is nearer to the number 2^5^ than 2^6^; we have

(21) $$\begin{array}{c} n_{0}=2^{5}+2^{3}+2=\left(2^{6}-2^{5}\right)+\left(2^{4}-2^{3}\right)+\left(2^{2}-2\right). \end{array}$$

Thus *i*_1_ = 6, *j*_1_ = 5, *i*_2_ = 4, *j*_2_ = 3, *i*_3_ = 2, and *j*_3_ = 1. And take *n*_0_ = 54 which is closer to the number 2^6^ than 2^5^; one obtains *n*_0_ = 2^6^ − 2^4^ + 6 = 2^6^ − 2^4^ + 2^3^ − 2. Then *i*_1_ = 6, *j*_1_ = 4, *i*_2_ = 3, and *j*_2_ = 1.

Consider Case 2 of *D*_0_. Denote

(22) $$\begin{array}{c} H\left(t\right)=a_{i_{t}}\left(\cdots \left(a_{i_{m-1}}\left(a_{i_{m}}H_{i_{m}}\cup D_{m-1}\right)\cdots \right)\cup D_{t}\right) \end{array}$$

for 1 < *t* ≤ *m*. Clearly, *ℋ*(*t*) ⊂ *H*_*j*_*t*−1__ and *ℋ*(1) = *D*_0_ in Case 2. For two factors *d*_1_ ∈ *ℋ*(*t*) and *d*_2_ ∈ *D*_*t*−1_, one has *d*_1_ *d*_2_ ∈ *D*_*t*−1_. Therefore,

(23) #d1,d2: d1∈Ht, d2∈Dt−1, d1d2=γ  =∑l=tm−12il−2jl+2im−1,γ∈Dt−1,0,γ∉Dt−1.

Specifically, if *t* = *m*, then

(24) #d1,d2: d1∈aimHim, d2∈Dm−1, d1d2=γ  =2im−1,γ∈Dm−1,0,γ∉Dm−1.

For *m* ≥ 1 and *γ* ∈ *H*_*i*_*m*__, there are 2^*i*_*m*_−1^ − 1 pairs of factors in *H*_*i*_*m*__, which each interaction is aliased with *γ*. Then

(25) B2aimHim,γ=B2Him,γ=2im−1−1,γ∈Him,0,γ∈Hq∖Him.

Based on the above results, we can obtain the value of *B*_2_(*D*_0_, *γ*) for any *γ* ∈ *H*_*q*_ in Case 2.

**Lemma 4 .**

Let *D*_0_ = *a*_*i*_1__(*a*_*i*_2__(⋯(*a*_*i*_*m*−1__(*a*_*i*_*m*__ *H*_*i*_*m*__ ∪ *D*_*m*−1_)⋯) ∪ *D*_2_) ∪ *D*_1_). Then

(26) B2D0,γ =cm2+2im−1−1,γ∈Him,cm−ct+2it2+2im−1,γ∈Dt,  t=1,…,m−1,ct2,γ∈Hjt−1∖Hit,  t=2,…,m,0,γ∈Hq∖Hi1,

where *c*(*t*) is defined in (14).

**Proof**

By (22), we obtain

(27) B2D0,γ =∑l=1m−1B2Dl,γ+B2aimHim,γ  +#d1,d2: d1∈aimHim, d2∈Dm−1, d1d2=γ  +∑l=2m−1#d1,d2: d1∈Hl, d2∈Dl−1, d1d2=γ.

For *γ* ∈ *H*_*q*_∖*H*_*i*_1__, we have *B*_2_(*D*_0_, *γ*) = 0. By Lemma 2, (25), and (23), analyze the following cases.

(i) For *γ* ∈ *H*_*i*_*m*__, we obtain
  (28) B2D0,γ=∑l=1m−1B2Dl,γ+B2aimHim,γ=∑l=1m−12il−1−2jl−1+2im−1−1.
(ii) For *γ* ∈ *D*_*t*_ with 1 ≤ *t* ≤ *m* − 1, one has
  (29) B2D0,γ =∑l=1t−1B2Dl,γ+B2Dt,γ  +#d1,d2: d1∈Ht+1, d2∈Dt, d1d2=γ =∑l=1t−12il−1−2jl−1  +∑l=tm−12il−2jl−2it−1+2im−1 =cm−ct+2it2+2im−1.
(iii) For *γ* ∈ *H*_*j*_*t*−1__∖*H*_*i*_*t*__ with 2 ≤ *t* ≤ *m*, *B*_2_(*D*_0_, *γ*) = ∑_*l*=1_^*t*−1^(2^*i*_*l*_−1^ − 2^*j*_*l*_−1^).

In Lemma 4, the value of *B*_2_(*D*_0_, *γ*) is relative to these pairs {*i*_*t*_, *j*_*t*_}_1<*t*<*m*_ and *i*_*m*_. For example, consider *n*_0_ = #{*D*_0_} = 21. Since *n*_0_ = (2^5^ − 2^4^)+(2^3^ − 2^2^) + 2 − 1, it yields *i*_1_ = 5, *j*_1_ = 4, *i*_2_ = 3, *j*_2_ = 2, and *i*_3_ = 1. Taking *n*_0_ = 29, we have *n*_0_ = 2^5^ − 3 = 2^5^ − 2^2^ + 1; thus *i*_1_ = 5, *j*_1_ = 2, and *i*_2_ = 1.

Lemmas 3 and 4, respectively, obtain the value of *B*_2_(*D*_0_, *γ*) that *D*_0_ consists of the last *n*_0_ columns of *T*_*r*_  (*r* ≤ *q*) for two cases. These results play a key role in calculating _1_^#^ *C*_2_ and _2_^#^ *C*_2_'s for all GMC 2^*n*−*m*^ designs with 5*N*/16 + 1 ≤ *n* ≤ *N* − 1. Next sections will, respectively, discuss two-level GMC designs with the factor number *n* satisfying (i) 5*N*/16 + 1 ≤ *n* < *N*/2 or (ii) *N*/2 ≤ *n* ≤ *N* − 1.

## 3. GMC 2^*n*−*m*^ Designs with 5*N*/16 + 1 ≤ *n* < *N*/2

Li et al. [10] showed all GMC 2^*n*−*m*^ designs with 5*N*/16 + 1 ≤ *n* < *N*/2, constructed by the last *n* columns of *T*_*q*_. In Section 2, *D*_0_ is constructed by Case 1 or Case 2, which is the last *n*_0_ columns of *T*_*r*_  (*r* ≤ *q*) for *n*_0_ = #{*D*_0_}. Therefore, for any GMC 2^*n*−*m*^ design *D* with 5*N*/16 + 1 ≤ *n* < *N*/2, its construction is similar to that of *D*_0_. In (3), take *j*_0_ = *r* = *q*.

**Theorem 5 .**

Consider GMC 2^*n*−*m*^ design

(30) $$\begin{array}{c} D=a_{i_{1}}\left(a_{i_{2}}\left(\cdots \left(a_{i_{m-1}}\left(a_{i_{m}}D_{m}\cup D_{m-1}\right)\cdots \right)\cup D_{2}\right)\cup D_{1}\right) \end{array}$$

with 5*N*/16 + 1 ≤ *n* < *N*/2. Then

(a) (31) C1#2kD=n,k=0,0,otherwise,
(b) (32) C2#2kD=n2jm−12,k=n2−1,n−ct+2it22it−2jt,k=n−ct+2it2−1,t=1,…,m,ct2jt−1−2it2,k=ct2−1,  t=2,…,m,0,otherwise,

where *c*(*t*) is defined in (14).

**Proof**

Evidently, *n* = ∑_*l*=1_^*m*^(2^*i*_*l*_^ − 2^*j*_*l*_^); we have *c*(*m* + 1) = *n*. By Lemma 3,

(33) B2D,γ =n2,γ∈Him,n−ct+2it2,γ∈Dt,  t=1,…,m,ct2,γ∈Hjt−1∖Hit,  t=2,…,m,0,γ∈Hq∖Hi1.

(a) Since *D* ⊂ *H*_*q*_∖*H*_*i*_1__, hence by (33) and (5)
  (34) C1#20D=#γ: γ∈D,B2D,γ=0=#D=n.

Otherwise, _1_^#^ *C*_2_^(*k*)^(*D*) = 0 for *k* ≠ 0.

(b) Following (33) and (6), we obtain
  (35) C2#2kD=k+1×#γ: γ∈Hjm,B2D,γ=k+1 +∑t=1m#γ: γ∈Dt,B2D,γ=k+1 +∑t=2m#γ: γ∈Hjt−1∖Hit,B2D,γ=k+1 +#γ: γ∈Hq∖Hi1,B2D,γ=k+1.

If *k* = *n*/2 − 1, then

(36) C2#2kD=n#Hjm2=n2jm−12.

For *k* = *n* − (*c*(*t*) + 2^*i*_*t*_^)/2 − 1 with 1 ≤ *t* ≤ *m*, one has

(37) C2#2kD=n−ct+2it2#Dt=n−ct+2it22it−2jt.

And if *k* = *c*(*t*)/2 − 1 with 1 < *t* ≤ *m*, then

(38) C2#2kD=ct#Hjt−1∖Hit2=ct2jt−1−2it2.

Otherwise, _2_^#^ *C*_2_^(*k*)^(*D*) = 0.

For GMC 2^*n*−*m*^ design with 5*N*/16 + 1 ≤ *n* < *N*/2, Theorem 5 reveals that the value of _1_^#^ *C*_2_ only depends on the factor number *n*. However, the value of _2_^#^ *C*_2_ is related to the numbers {*i*_*t*_, *j*_*t*_}_1≤*t*≤*m*_ besides *n*. We illustrate them via a simple example.

**Example 6 .**

Take *q* = 5 and *n* = 10; consider GMC 2^10−5^ design *D*. Since *n* = 2^3^ + 2 = (2^4^ − 2^3^)+(2^2^ − 2), clearly, we have *i*_1_ = 4, *j*_1_ = 3, *i*_2_ = 2, and *j*_2_ = 1. Hence, 2^*i*_1_^ − 2^*j*_1_^ = 8, 2^*i*_2_^ − 2^*j*_2_^ = 2, 2^*j*_1_^ − 2^*i*_2_^ = 4, and *c*(1) = 0, *c*(2) = 8. By Theorem 5, we get

(39) C1#2kD=10,k=0,0,otherwise,C2#2kD=16,k=1,24,k=3,5,k=4,0,otherwise.

Theorem 5 applies to the case that the factor number *n* of GMC design is even. If *n* is odd, similar to the proof of Theorem 5, by Lemma 4, one can get the result below.

**Theorem 7 .**

Consider GMC 2^*n*−*m*^ design

(40) $$\begin{array}{c} D=a_{i_{1}}\left(a_{i_{2}}\left(\cdots \left(a_{i_{m-1}}\left(a_{i_{m}}H_{i_{m}}\cup D_{m-1}\right)\cdots \right)\cup D_{2}\right)\cup D_{1}\right) \end{array}$$

with 5*N*/16 + 1 ≤ *n* < *N*/2. Then

(a) (41) C1#2kD=n,k=0,0,otherwise,
(b) (42) C2#2kD=n−12im−12,k=n−12−1,n−ct+2it22it−2jt,k=n−ct+2it2−1,t=1,…,m−1,ct2jt−1−2it2,k=ct2−1,  t=2,…,m,0,otherwise,

where *c*(*t*) is defined in (14).

**Proof**

Note that *n* = ∑_*l*=1_^*m*−1^(2^*i*_*l*_^ − 2^*j*_*l*_^) + 2^*i*_*m*_^ − 1 = *c*(*m*) + 2^*i*_*m*_^ − 1.

**Example 8 .**

Let *q* = 5 and *n* = 11; consider GMC 2^11−6^ design *D*. Here *n* = 2^4^ − 2^3^ + 2^2^ − 1; we have *i*_1_ = 4, *j*_1_ = 3, and *i*_2_ = 2. Thus, *c*(2) = 2^*i*_1_^ − 2^*j*_1_^ = 8 and 2^*j*_1_^ − 2^*i*_2_^ = 4. Following Theorem 7, it is directly obtained by

(43) C1#2kD=11,k=0,0,otherwise,C2#2kD=24,k=2,16,k=3,15,k=4,0,otherwise.

## 4. GMC 2^*n*−*m*^ Designs with *N*/2 ≤ *n* ≤ *N* − 1

In Section 2, we know that any GMC 2^*n*−*m*^ design with *N*/2 ≤ *n* ≤ *N* − 1 is constructed by *D*_0_ ∪ *S*_*qr*_, where *D*_0_ is the last *n* − (*N* − 2^*r*^) columns of *T*_*r*_  (*r* < *q*). Lemmas 3 and 4 have shown the confounding information of *D*_0_. Next we will study a special design *S*_*qr*_ = *H*_*q*_∖*H*_*r*_  (*r* < *q*), which consists of the last *N* − 2^*r*^ columns of *H*_*q*_. Since *r* < *q*, the factor number of the design *S*_*qr*_ satisfies *N* − 2^*r*^ ≥ *N*/2. Hence, the design *S*_*qr*_ has GMC. By Lemma 2, we directly give the value of *B*_2_(*S*_*qr*_, *γ*) as follows:

(44) $$\begin{array}{c} B_{2}\left(S_{qr},\gamma \right)=\left\{\begin{array}{cc} \frac{N}{2}-2^{r}, & \gamma \in H_{q}\setminus H_{r},\\ \frac{N}{2}-2^{r-1}, & \gamma \in H_{r}. \end{array}\right. \end{array}$$

Next we discuss the values of _1_^#^ *C*_2_ and _2_^#^ *C*_2_ for GMC design *S*_*qr*_ with *r* < *q*.

**Theorem 9 .**

Consider any GMC design *S*_*qr*_ = *H*_*q*_∖*H*_*r*_ for *r* < *q*. Then

(a) (45) C1#2kSqr=N−2r,k=N2−2r,0,otherwise,
(b) (46) C2#2kSqr=N2−2rN−2r,k=N2−2r−1,N2−2r−12r−1,k=N2−2r−1−1,0,otherwise.

**Proof**

(a) If *k* = *N*/2 − 2^*r*^, by (44), then

(47) C1#2kSqr=#γ: γ∈Sqr,B2Sqr,γ=k=#Sqr=N−2r.

Otherwise, _1_^#^ *C*_2_^(*k*)^(*S*_*qr*_) = 0.

(b) For *k* ≥ 0, note that
  (48) C2#2kSqr=k+1×#γ: γ∈Sqr,B2Sqr,γ=k+1 +#γ: γ∈Hr,B2Sqr,γ=k+1.

If *k* = *N*/2 − 2^*r*^ − 1, thus by (44)

(49) C2#2kSqr=N2−2r#Sqr=N2−2rN−2r.

Similarly, for *k* = *N*/2 − 2^*r*−1^ − 1, we have

(50) C2#2kSqr=N2−2r−1#Hr=N2−2r−12r−1.

For GMC design *S*_*qr*_  (*r* < *q*), the values of _1_^#^ *C*_2_ and _2_^#^ *C*_2_ only rely on two numbers *N* and *r*. In particular, if *r* = *q* − 1, then *S*_*q*(*q*−1)_ = *T*_*q*_. By Theorem 9, one has

(51) C1#2kTq=N2,k=0,0,k≠0,C2#2kTq=N4N/2−1,k=N4−1,0,k≠N4−1.

The next example is used to illustrate this above result.

**Example 10 .**

Consider GMC 2^16−11^ design *S*_54_. Since *r* = 4 and *N* = 32, one directly gets

(52) C1#2kD=16,k=0,0,k≠0,C2#2kD=120,k=7,0,k≠7.

On the other hand, every GMC 2^*n*−*m*^ design *D* with *n* ≥ *N*/2 can be constructed by the form (*D*∖*S*_*qr*_) ∪ *S*_*qr*_, where *D*∖*S*_*qr*_ consists of the last *n* − (*N* − 2^*r*^) columns of *T*_*r*_. Then, *D*_0_ = *D*∖*S*_*qr*_. Based on Lemma  3 of Li et al. [10], we obtain the relationship of *D* and *D*_0_ as follows:

(53) $$\begin{array}{c} B_{2}\left(D,\gamma \right)=\left\{\begin{array}{cc} n-\frac{N}{2}, & \gamma \in H_{q}\setminus H_{r},\\ B_{2}\left(D_{0},\gamma \right)+\frac{N}{2}-2^{r-1}, & \gamma \in H_{r}. \end{array}\right. \end{array}$$

Therefore, we can get the following result.

**Theorem 11 .**

Consider GMC 2^*n*−*m*^ design *D* = *D*_0_ ∪ *S*_*qr*_ with *r* < *q*, where *D*_0_ = *a*_*i*_1__(*a*_*i*_2__(⋯(*a*_*i*_*m*−1__(*a*_*i*_*m*__ *D*_*m*_ ∪ *D*_*m*−1_)⋯) ∪ *D*_2_) ∪ *D*_1_). Then

(a) (54) $$\begin{array}{c} B_{2}\left(D,\gamma \right)=\left\{\begin{array}{cc} \frac{n}{2}, & \gamma \in H_{j_{m}},\\ n-\frac{\left(a\left(t\right)+2^{i_{t}}\right)}{2}, & \gamma \in D_{t},\;\;t=1,\ldots ,m,\\ \frac{a\left(t\right)}{2}, & \gamma \in H_{j_{t-1}}\setminus H_{i_{t}},\;\;t=2,\ldots ,m,\\ \frac{N}{2}-2^{r-1}, & \gamma \in H_{r}\setminus H_{i_{1}},\\ n-\frac{N}{2}, & \gamma \in H_{q}\setminus H_{r}, \end{array}\right. \end{array}$$
(b) (55) C1#2kD=N−2r,k=n−N2,n−N+2r,k=N2−2r−1,0,otherwise,
(c) (56) C2#2kD=n2jm−12,k=n2−1,n−at+2it22it−2jt,k=n−at+2it2−1,t=1,…,m,at2jt−1−2it2,k=at2−1,t=2,…,m,N2−2r−12r−2i1,k=N2−2r−1−1,n−N2N−2r,k=n−N2−1,0,otherwise,

where *a*(*t*) = *N* − 2^*r*^ + *c*(*t*) and *c*(*t*) is defined in (14).

**Proof**

(a) By (53) and Lemma 3, note that

(57) $$\begin{array}{c} c\left(m+1\right)=\#\left\{D_{0}\right\}=\overset{m}{\underset{l=1}{\sum }}\left(2^{i_{l}}-2^{j_{l}}\right)=n-N+2^{r} \end{array}$$

yields (a).

(b) For *γ* ∈ *S*_*qr*_, by (a), *B*_2_(*D*, *γ*) = *n* − *N*/2. If *k* = *n* − *N*/2, then
  (58) C1#2kD=#Sqr=N−2r.

Since *D*_0_ ⊂ *H*_*r*_∖*H*_*i*_1__, for *k* = *N*/2 − 2^*r*−1^, we have

(59) C1#2kD=#D0=#⋃t=1mDt=∑t=1m2it−2jt=n−N+2r.

(c) Since
  (60) C2#2kD=k+1×#γ: γ∈Hjm,B2D,γ=k+1 +∑t=1m#γ: γ∈Dl,B2D,γ=k+1 +∑t=2m#γ: γ∈Hjt−1∖Hik,B2D,γ=k+1 +#γ: γ∈Hr∖Hi1,B2D,γ=k+1 +#γ: γ∈Hq∖Hr,B2D,γ=k+1,

by (a), the result follows.

When the factor number *n* of a GMC design satisfying *N*/2 ≤ *n* ≤ *N* − 1 is even, by Theorem 11, we obtain values of the corresponding _1_^#^ *C*_2_ and _1_^#^ *C*_2_. The next example illustrates this point.

**Example 12 .**

Let *q* = 8, *r* = 7; consider GMC 2^154−146^ design *D* = *D*_0_ ∪ *S*_87_. Since *n*_0_ = #{*D*_0_} = 26 and

(61) $$\begin{array}{c} n_{0}=2^{5}-6=\left(2^{5}-2^{3}\right)+\left(2^{2}-2\right), \end{array}$$

we have *i*_1_ = 5, *j*_1_ = 3, *i*_2_ = 2, and *j*_2_ = 1. Thus, 2^*i*_1_^ − 2^*j*_1_^ = 24, 2^*i*_2_^ − 2^*j*_2_^ = 2 and *a*(1) = *N* − 2^*r*^ + *c*(1) = 128, *a*(2) = *N* − 2^*r*^ + *c*(2) = 152. By (b) and (c) of Theorem 11, one obtains

(62) C1#2kD=128,k=26,26,k=64,0,otherwise,C2#2kD=3328,k=25,6144,k=63,1776,k=73,456,k=75,77k=76,0,otherwise.

**Theorem 13 .**

Consider GMC 2^*n*−*m*^ design *D* = *D*_0_ ∪ *S*_*qr*_ with *r* < *q*, where *D*_0_ = *a*_*i*_1__(*a*_*i*_2__(⋯(*a*_*i*_*m*−1__(*a*_*i*_*m*__ *H*_*i*_*m*__ ∪ *D*_*m*−1_)⋯) ∪ *D*_2_) ∪ *D*_1_). Then

(a) (63) B2D,γ =n−12,γ∈Him,n−at+2it2,γ∈Dt,  t=1,…,m−1,at2,γ∈Hjt−1∖Hit,  t=2,…,m,N2−2r−1,γ∈Hr∖Hi1,n−N2,γ∈Hq∖Hr,
(b) (64) C1#2kD=N−2r,k=n−N2,n−N+2r,k=N2−2r−1,0,otherwise,
(c) (65) C2#2kD=n−12im−12,k=n−12−1,n−at+2it22it−2jt,k=n−at+2it2−1,t=1,…,m−1,at2jt−1−2it2,k=at2−1,t=2,…,m,N2−2r−12r−2i1,k=N2−2r−1−1,n−N2N−2r,k=n−N2−1,0,otherwise,

where *a*(*t*) = *N* − 2^*r*^ + *c*(*t*) and *c*(*t*) is defined in (14).

**Proof**

Only prove (a). Since

(66) $$\begin{array}{c} \#\left\{D_{0}\right\}=\overset{m-1}{\underset{l=1}{\sum }}\left(2^{i_{l}}-2^{j_{l}}\right)+2^{i_{m}}-1=n-N+2^{r}, \end{array}$$

one has *c*(*m*) = *n* − (*N* − 2^*r*^)−(2^*i*_*m*_^ − 1). By (53) and Lemma 4, yields (a).

The proof of (b) and (c) is similar to those of Theorem 11. The following example serves to show its application.

**Example 14 .**

Let *q* = 8, *r* = 7, and *N* = 256 and consider GMC 2^135−127^ design *D* = *D*_0_ ∪ *S*_87_. Since #{*D*_0_} = 2^3^ − 1, we have *i*_1_ = 3. By (b) and (c) of Theorem 13, one gets

(67) C1#2kD=128,k=7,7,k=64,0,otherwise,C2#2kD=896,k=6,7680,k=63,469,k=66,0,otherwise.

## 5. Concluding Remark

Based on construction of GMC 2^*n*−*m*^ designs with 5*N*/16 + 1 ≤ *n* ≤ *N* − 1, we obtain the mathematical formulation to calculate the values of _1_^#^ *C*_2_ and _2_^#^ *C*_2_ in the AENP. These results are very useful to analyze the confounding information among lower-order factors of two-level GMC designs. For GMC 2^*n*−*m*^ designs satisfying *n* ∉ [5*N*/16 + 1, *N* − 1], some further studies in this direction are in progress.
